# Low Muscle Mass Is Associated with Readmission for Inflammatory Bowel Disease

**DOI:** 10.5152/tjg.2022.21711

**Published:** 2023-02-01

**Authors:** Sifan Liu, Zibin Tian, Yueping Jiang, Xueli Ding, Shengbo Jin, Xue Jing

**Affiliations:** Department of Gastroenterology, the Affiliated Hospital of Qingdao University, Qingdao, Shandong Province, China

**Keywords:** Crohn’s disease, inflammatory bowel disease, muscle, nutrition, ulcerative colitis

## Abstract

**Background:**

Patients with inflammatory bowel disease tend to have malnutrition, frailty, and low muscle mass, which impact on poor clinical outcomes. Abdominal computed tomography is frequently used to assess body composition. This study aimed to evaluate the association of low muscle mass and readmission within 1 year in patients with inflammatory bowel disease during hospitalization and follow-up.

**Methods:**

A total of 211 patients with inflammatory bowel disease who had undergone computed tomography scans were included retrospectively. They were divided into subgroups based on disease activity. The male patients with skeletal muscle index ≤45.4 cm^2^/m^2^ and the female patients with skeletal muscle index ≤ 34.3 cm^2^/m^2^ were considered to have low muscle mass. Sociodemographic, clinical, and prognostic data were recorded. The analyses were done using the Statistical Package for the Social Sciences 25.0 software.

**Results:**

The prevalence rate of low muscle mass was 64.7%. Low body mass index and hemoglobin, high erythrocyte sedimentation rate, smoking, and gastrointestinal surgery history were risk factors for low muscle mass (*P *< .05). Patients using steroids and biologics and using them more than 7 months were prone to develop low muscle mass and readmission (*P *< .05), while patients using immunomodulators were not. Inflammatory bowel disease patients with visceral fat area/subcutaneous fat area ≥0.71 were likely to readmit within 1 year than those with visceral fat area/subcutaneous fat area <0.71 (*P *< .05). Overweight or obese inflammatory bowel disease patients with low muscle mass had a shorter time to readmission than those without low muscle mass (*P *< .05).

**Conclusions:**

Overweight/obese inflammatory bowel disease patients with low muscle mass and patients using steroids and biologics have shorter time to readmission within 1 year regardless of disease activity.

**Main Points**

- Low muscle mass is common in inflammatory bowel disease (IBD) patients.
- Most patients (96%) with IBD self-selected their diets.
- Inflammatory bowel disease patients with low muscle mass had higher C-reactive protein and erythrocyte sedimentation rate and lower albumin than those without low muscle mass.
- The use of steroids, biologics, and low muscle mass is a vicious cycle: IBD patients with low muscle mass tend to use more steroids and biologics. The use of steroids and biologics and the average duration of use more than 7 months are risk factors for IBD patients developing low muscle mass. Inflammatory bowel disease patients with low muscle mass were prone to readmission within 1 year regardless of disease activity.
- Overweight or obese IBD patients with low muscle mass had a shorter time to readmission than those without low muscle mass. The cutoff point of visceral fat area/subcutaneous fat area was 0.71.

## Introduction

Patients with inflammatory bowel disease (IBD), including ulcerative colitis (UC) and Crohn’s disease (CD), have altered body composition, such as diminished muscle mass, protein depletion, and higher abdominal adiposity.^1^ These changes are due to a combination of inadequate intake, poor absorption, excessive loss, and increased demand for nutrients.^2^ The changes in body composition may lead to malnutrition, low muscle mass, and unfavorable prognosis, including the need for surgery, parenteral manifestations, and delayed clinical remission.^3-4^ In adult IBD patients, the prevalence of low muscle mass was around 45%,^1^ and it was around 93.6% in pediatric patients.^5,6^ Low muscle mass has been shown to be related to high morbidity, mortality, and poor quality of life.^7-9^

There were multiple methods to assess body composition such as abdominal computed tomography (CT), magnetic resonance, and dual energy x-ray absorptiometry.^1^ Abdominal CT can be used to accurately estimate and assess body composition and is frequently used in clinics among IBD patients.^1,2^ Muscle mass at L3 vertebra through abdominal CT is associated with the whole-body muscle mass strongly. In this study, we used skeletal muscle mass at L3 vertebra level through abdominal CT to assess low muscle mass.

The relationship between low muscle mass and readmission within 1 year is not sufficiently understood. Hence, in this study, we aimed to evaluate the association of low muscle mass and combined low muscle mass and visceral obesity and the influence of using steroids or biologics or immunomodulators on readmission within 1 year in patients with IBD.

## Materials and Methods

This study was registered on COLCOT Clinical Trials.gov.com with the number NCT ChiCTR2100045179 and approved by the Ethics Committee of the Affiliated Hospital of Qingdao University (approval no. QYFY WZLL 26290).

### Study Population

The study participants included patients with IBD admitted to our hospital from January 1, 2010, to July 31, 2020. The diagnosis of IBD included clinical, hematological, endoscopic, and pathological examination. All the patients agreed to participate in the study and signed informed consent. The eligible age for the inclusion in the study was between 18 and 60 years. The exclusion criteria were as follows: (1) presence of comorbidities, such as cancer, liver diseases, and severe organ insufficiency, which may reduce the potential nutritional status and (2) incomplete abdominal CT results.

### Data Collection

**Figure 1.**
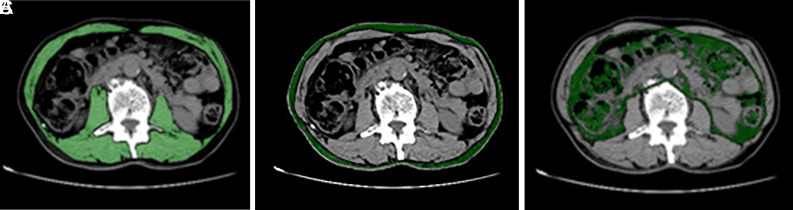
Skeletal muscle area measurement range (−30 to 150 HU). (A), SFA measurement range (−190 to −30 HU) (B), and VFA measurement range (−190 to −30 HU), (C) show body composition measurement on abdominal CT (at the level of the third lumbar vertebra). SFA, subcutaneous fat area; VFA, visceral fat area; CT, computed tomography; HU, Hounsfield unit.

The patients’ sociodemographic, clinical, prognostic, laboratory, and abdominal CT results were recorded. Ulcerative colitis patients were divided into 3 subgroups based on the Modified Mayo Endoscopic Score (MMES) related with disease activity.^10^: mild group (3-5 score), moderate group (6-10 score), and severe group (11-12 score). Crohn’s disease patients were divided into these subgroups according to best CDAI score related with disease activity.^11^: mild group (150-220 score), moderate group (221-450 score), and severe group (>450 score). The skeletal muscle area (SMA), subcutaneous fat area (SFA), and visceral fat area (VFA) were recorded through abdominal CT as shown in Figure 1. Skeletal muscle index (SMI), defined as SMA at the level of the third lumbar vertebra on CT divided by the square of the height, was used to diagnose low muscle mass. Male IBD patients with SMI ≤45.4 cm^2^/m^2^ and female IBD patients with SMI ≤34.3 cm^2^/m^2^ were considered to have low muscle mass.^12^ Meanwhile, the IBD questionnaire (IBDQ)^13^ was done among these patients to understand their attitudes to the influence caused by IBD in their life.

The outcomes included the use of steroids or biologics or immunomodulators within 1 year and the time to readmission within 1 year due to disease progression.

### Statistical Analysis

Quantitative variables were presented as mean ± standard deviation, if normally distributed, or as median and interquartile range, if not normally distributed. Qualitative variables were presented as numbers or percentages. The chi-square test was used to compare categorical variables. Student’s *t*-test, Mann–Whitney *U* test, or Wilcoxon rank sum test were used for quantitative variables. The Kaplan–Meier method was used for survival analysis, and log-rank method was used for comparison between groups, with *P* < .05 considered statistically significant. The odds ratio (OR) and 95% CIs were also shown in the analysis. Univariate and multivariate analysis were done for risk factors with *P *< .05 considered significantly different. The analysis software was Statistical Package for the Social Sciences 25.0 (IBM Corp.; Armonk, NY, USA).

## Results

### Clinic Characteristics Between Low Muscle Mass and Without Low Muscle Mass Groups

A total of 211 patients with IBD were enrolled and 142 (67.3%) of them with UC and 69 (32.7%) with CD. The average age of the patients was 45.3 years. In UC patients, there were 34 patients in the mild group, 59 patients in the moderate group, and 49 patients in the severe group. Among CD patients, 17 patients were in the mild group, 22 patients were in the moderate group, and 30 patients were in the severe group. Based on CT measurements at the level of the third lumbar vertebra, more than half of the patients (56.4%) had low muscle mass, including 72 patients with UC and 47 patients with CD. Inflammatory bowel disease patients with low muscle mass tend to have lower body mass index (BMI) (*P *< .001), less IBD-related complications (*P *= .033), lower albumin (alb) (*P *< .001), lower fasting blood glucose (*P *= .025), lower total triglycerides (TG) (*P *= .013) and more smoking history (*P *= .001), more gastrointestinal surgery history (*P *< .001), more parenteral manifestations (*P *< .001), higher total cholesterol (TC) (*P *< .001), higher low-density lipoprotein (*P *= .021), higher hemoglobin (Hb) (*P *< .001), higher C-reactive protein (CRP) (*P *< .001), higher erythrocyte sedimentation rate (ESR) (*P *< .001), and more of them were diagnosed as CD (*P *= .017) than those without low muscle mass (Table 1).

### Risk Factors of Inflammatory Bowel Disease Patients Developing Low Muscle Mass

An univariate logistic regression was done based on BMI, smoking history, gastrointestinal surgery history, parenteral manifestations, IBD-related complications, diagnosis of UC or CD, alb, fasting blood glucose, TC, TG, HDL, Hb, CRP, and ESR. Factors with *P* value <.05 were considered significantly different and came to a multivariate logistic regression. The multivariate logistic regression showed lower BMI (*B *= –0.242, *P *= .007), lower Hb (*B *= –0.029, *P *= .011), higher ESR (*B *= 0.062, *P *= .020), more smoking history (*B *= 1.507, *P *= .010), and more gastrointestinal surgery history (*B *= 1.315, *P *= .017) were risk factors for IBD patients developing low muscle mass (Table 2).

### Dietary Intentions of Inflammatory Bowel Disease Patients

A total of 202 patients (96%) including 80 UC and 122 CD chose to avoid eating several foods based on individual factors, such as bean products, meat, eggs, fruits, and fish, as shown by the IBDQ questionnaire.

### Use of Steroids, Biologics, and Immunomodulators in Inflammatory Bowel Disease Patients

A total of 62 patients used steroids including methylprednisolone and prednisone, 35 patients used biologics such as remicade and adalimumab, and 10 patients used immunomodulators such as thalidomide and azathioprine within 1 year. The median time of using steroids was 7 months within 1 year, as same as biologics, and immunomodulators was 6 months within 1 year.

### Steroids, Biologics, Immunomodulators and Low Muscle Mass in Inflammatory Bowel Disease Patients

Inflammatory bowel disease patients with low muscle mass were more likely to use steroids (47.1% vs 6.5%, *P *< .001) and biologics (25.2% vs 5.4%, *P *< .001), while not immunomodulatory (6.7% vs 2.2%, *P *= .192) than those without low muscle mass.

Subsequently, we did a univariate analysis based on whether patients used steroids or biologics or immunomodulators were correlated with low muscle mass. The results showed IBD patients used steroids (*P *< .001) or biologics (*P *< .001) and those who use steroids > 7 months (*P *= .008) and biologics > 7 months (*P *= .027) within 1 year were prone to low muscle mass when compared to patients who use immunomodulators (*P *= .143) and using it >6 months (*P *= .236). Then we did a multivariate logistic regression, and the results showed that the use of steroids (*P *< .001) and the use of biologics (*P *= .019) within 1 year were risk factors for IBD patients developing low muscle mass (Table 3).

The same analysis was done with readmission within 1 year. As shown in Table 4, IBD patients using steroids (*P *< .001) or biologics (*P *= .020) and using steroids > 7 months (*P *= .007) and biologics > 7 months (*P *= .008) were prone to readmit within 1 year while patients using immunomodulators (*P *= .270) and using it > 6 months (*P *= .107) were not prone to readmit. The use of steroids (*P *< .001) and the use of biologics (*P *< .001) were risk factors for IBD patients readmitting within 1 year.

### SMA, VFA, SFA, VFA/SFA, and Readmission Within 1 Year in IBD Patients

**Figure 2.**
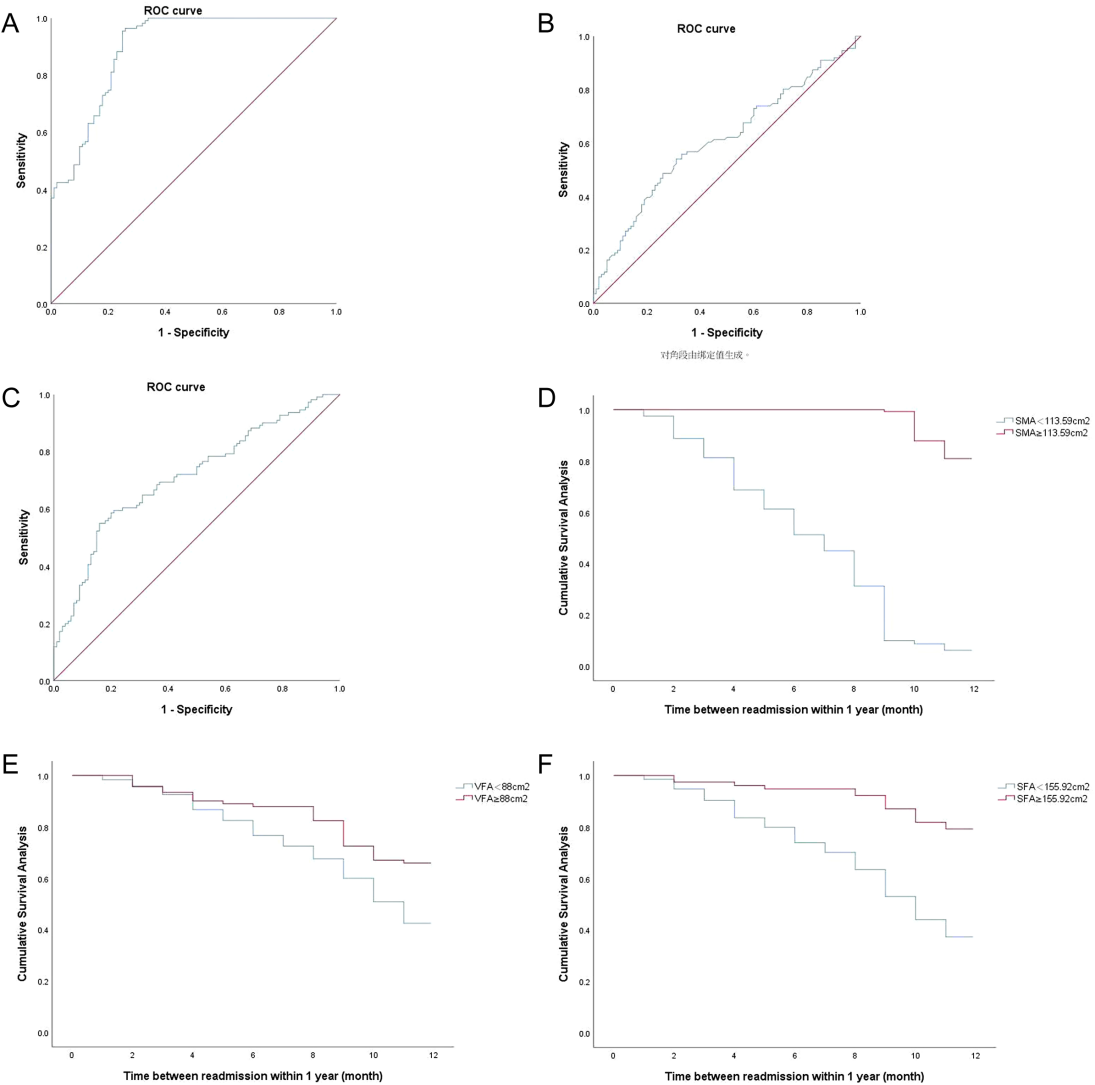
ROC curves of SMA (A), VFA (B), SFA (C), and time to readmission within 1 year. Cumulative survival analysis between in the high group and low group of SMA (D), VFA (E), SFA (F), and time between readmission within 1 year. SMA, skeletal muscle area; VFA, visceral fat area; SFA, subcutaneous fat area.

A total of 77 of 119 IBD patients with low muscle mass (64.7%) were readmitted, in contrast to only 23 of 92 (25.0%) without low muscle mass (*P *< .05). In addition, we calculated SMA, VFA, and SFA through CT at the level of the third lumbar vertebra. Receiver Operating Characteristic (ROC) curves were created to evaluate the relationship of SMA, VFA, SFA, and readmission within 1 year. For SMA, the area under ROC curve was 0.896, with a significance level of *P *= .022 and the cutoff value was 113.59 cm^2^, as shown in Figure 2A. For VFA, the area under ROC curve was 0.609, with a significance level of *P *= .039 and the cutoff value of 88 cm^2^ (Figure 2B). For SFA, the area under ROC curve was 0.711, with a significance level of *P *= .035 and the cutoff value of 155.92 cm^2^, as shown in Figure 2C. Then, we did Kaplan–Meier analysis and log-rank tests between the high group and low group of SMA, VFA, and SFA according to the above cut-off values with readmission within 1 year. The results showed IBD patients whose SMA< 113.59 cm^2^ or VFA< 88 cm^2^ or SFA< 155.92 cm^2^ were more likely to readmit within 1 year (Figure 2D-2F).

**Figure 3.**
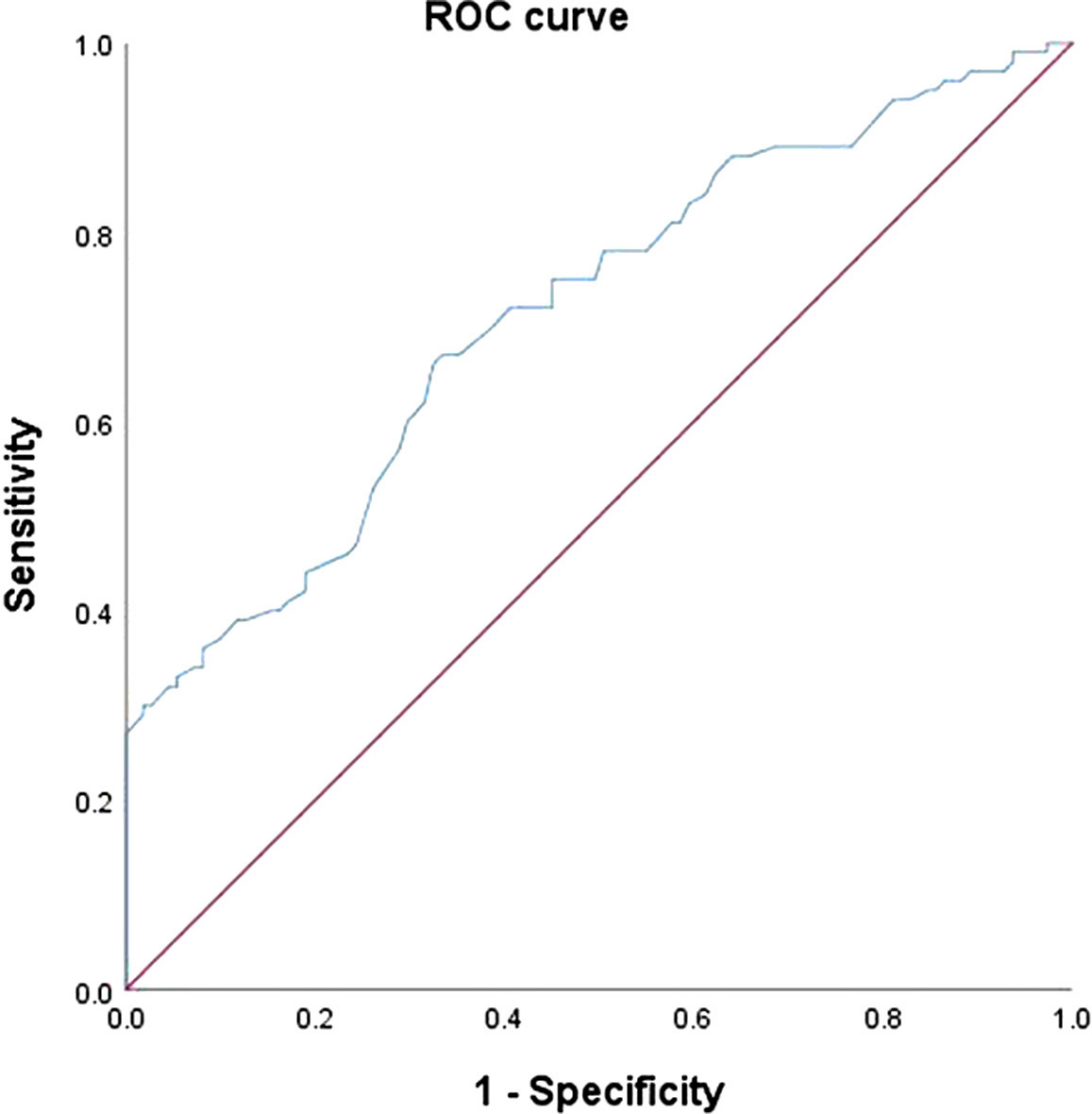
ROC curves of VFA/SFA and time to readmission within 1 year. VFA, visceral fat area; SFA, visceral fat area.

Then, the ratio of VFA and SFA (VFA/SFA) was calculated to further clarify the relationship between visceral fat and subcutaneous fat and prognosis. An ROC curve was created to evaluate its predictive value for the time to readmission within 1 year. As shown in Figure 3, the area under the curve was 0.713, with a significance level of *P *< .001 and the cutoff value of 0.71. Hence, IBD patients with VFA/SFA ≥0.71 were more likely to be readmitted within 1 year than those with VFA/SFA <0.71.

### Disease Activity and Readmission Within 1 Year

Ulcerative colitis and CD patients were divided into mild, moderate, and severe subgroups according to MMES and best CDAI score. The chi-square test was used to verify whether low muscle mass was associated with readmission within 1 year regardless of disease activity. The result show that the patients with low muscle mass were prone to readmit within 1 year in UC patients in mild (*P *= .027), moderate (*P *= .047), and severe (*P *< .001) groups. Similarly, in CD patients, patients with low muscle mass were more likely to readmit within 1 year in mild (*P *< .001), moderate (*P *< .001), and severe (*P *< .001) groups (Table 5). Inflammatory bowel disease patients with low muscle mass were prone to readmit within 1 year without associated disease severity.

### Overweight or Obese Inflammatory Bowel Disease Patients and Readmission Within 1 Year

Traditionally, BMI was used to measure patients’ nutritional status. In this study, there were 103 underweight patients, 39 with normal body weight, and 69 overweight or obese patients in general. The chi-square test was used to compare the readmission within 1 year between these groups. The results showed that overweight or obese patients were readmitted more commonly than underweight or normal ones (*P *< .001).

**Figure 4.**
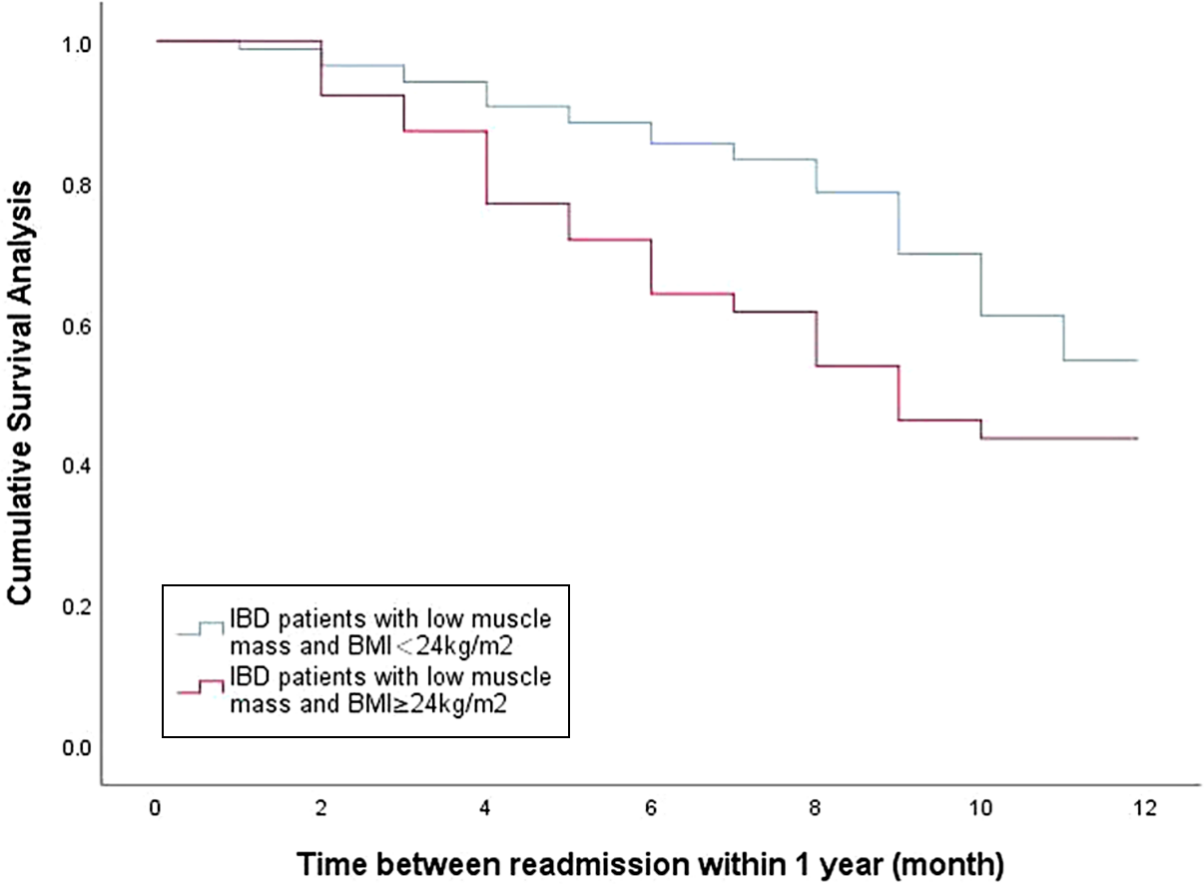
Cumulative survival analysis between IBD patients with low muscle mass whose BMI<24 kg/m^2^ or ≥24 kg/m^2^ and time between readmission within 1 year. IBD, inflammatory bowel disease; BMI, body mass index.

Overweight or obese patients with low muscle mass were those IBD patients with BMI >24 kg/m^2^ and low muscle mass.^1^ In this study, 39 IBD patients with low muscle mass were overweight or obese, including 35 patients with UC and 4 patients with CD. The Kaplan–Meier analysis and log-rank test were applied between overweight or obese patients with low muscle mass and overweight or obese IBD patients without low muscle mass (Figure 4). The results showed that overweight or obese IBD patients with low muscle mass had a shorter time to readmission than overweight or obese patients without low muscle mass (8.4 months vs. 10.0 months, *P *= .044).

## Discussion

This study showed that low muscle mass as assessed by CT was associated with readmission for IBD. Previous studies have shown that the prevalence of low muscle mass in IBD patients ranges between 40% and 60%.^1,14-17^ In this study, the prevalence of low muscle mass was 56.4%, which is consistent with the previous studies, and indicates that low muscle mass is common among IBD patients. Skeletal muscle index at the L3 level was used to evaluate muscle mass. There are different cut-off values from United States,^12^ Australia,^18^ Japan,^19^ Holland^20^, and Turkish scientists in 2021,^21^ due to regional diet and living habits and even geographical differences.

For IBD patients, self-imposed food restriction behavior to prevent a disease outbreak,^2^ malabsorption, excessive loss of nutrients, and increased demand for nutrients may cause alterations in body composition, malnutrition, and low muscle mass, which have a negative effect on prognosis; namely, they experience a higher proportion of complications after operations, longer hospital stays and higher costs, and decreased quality of life.^6,7,22,23^ In our study, around 96% IBD patients chose to avoid eating some kinds of foods they considered harmful to their health.

Previous studies have shown that the skeletal muscle volume is associated with not only age but also BMI, serum CRP, and ESR—indicators of chronic malnutrition or inflammation status of IBD patients.^1,15,23^ Likewise, in this study, SMI correlated positively with CRP and ESR, smoking history, gastrointestinal surgery history and negatively with BMI and Hb. Smoking is harmful to lung, blood vessels and may cause a delay of intestinal mucosa healing, which lead to nutrition absorption disorder. Having an intestinal surgery history before reminds severe diseases. In clinical settings, CRP and ESR are considered inflammatory serum markers. Inflammatory bowel disease patients with low SMI were more likely to have low muscle mass; they also had high CRP and ESR, reflecting the serious chronic and persistent inflammation. Low BMI and Hb remind a bad nutritional status, which may cause low muscle mass as well.

Inflammatory bowel disease patients who use steroids or biologics or immunomodulators and the longer time of using them reflect severe disease status, which may cause low muscle mass and readmission.^24,25^ In this study, the use of steroids and biologics and the average duration of use them more than 7 months are associated with low muscle mass and readmission while the use of immunomodulators was not associated. Hence, it is important to pay attention to IBD patients who use steroids or biologics in case of poor outcomes. Inflammatory bowel disease patients can be divided into mild, moderate, and severe subgroups based on disease activity. The use of steroids and biologics, low muscle mass, and readmission within 1 year were expected to exist in high disease activity. Hence, we did a chi-square test to verify whether low muscle mass was associated with readmission within 1 year regardless of disease activity. The results show UC or CD patients with low muscle mass were prone to readmit within 1 year regardless of disease activity, which imply low muscle mass can predict readmission within 1 year forcefully. Therefore, paying attention to low muscle mass and its effect on readmission within 1 year, regardless of disease activity, is necessary and helpful to IBD patients.

In this study, patients with low muscle mass, especially those with VFA/SFA ≥0.71 and those who were overweight or obese, had poorer clinical outcomes, as reflected both in the use of steroids or biologics or immunomodulators and in the shorter time to readmission within 1 year. Muscle loss and high visceral fat were the risk factors for these outcomes, which is consistent with previous studies.^1,26-28^ As a chronic systemic autoimmune disease that involves inflammation in digestive tract, IBD can affect the quality of life.^23^ Early detection and intervention of low muscle mass can delay or even reverse the occurrence of poor prognosis. Hence, it is necessary to bring the detection of low muscle mass into clinical nutrition assessment and develop individualized therapeutic strategies in order to improve the prognosis of patients with IBD.

There are some limitations in this study. First, it was a single-center study, which cannot represent most IBD patients. Second, only muscle area was examined, but not muscle function, which may have introduced certain bias. Therefore, multicenter, prospective, and more comprehensive studies are needed.

## Conclusions

Low muscle mass is common in IBD patients. Overweight/obese IBD patients with low muscle mass and using steroids and biologics, not immunomodulators, have shorter time to readmission within 1 year regardless of disease severity. It is suggested that clinicians should strengthen the screening of low muscle mass and pay attention to nutritional support for IBD patients.

## Figures and Tables

**Table 1.** Demographic Data in IBD Patients with Low Muscle Mass and Without Low Muscle Mass

	Low Muscle Mass (n)	Without Low Muscle Mass (n)	*P*
Sex			
Male	74	48	.094
Female	45	44	
Age (years)	46 ± 13.9	49±12.4	.095
BMI (kg/m^2^)	20.8 ± 3.2	23.9 ± 3.1	<.001*
Medical history (months)	28 ± 73	28 ± 74	.376
Smoking history	77 (64.71%)	38 (41.30%)	.001*
Drinking history	67 (56.30%)	41 (44.57%)	.098
Gastrointestinal surgery history	74 (62.18%)	32 (34.78%)	<.001*
Parenteral manifestations	81 (68.07%)	42 (45.65%)	<.001*
IBD-related complications	39 (32.77%)	44 (47.83%)	.033*
Diagnosis			
UC	72	70	.017*
CD^§^	47	22	
UC			
E1	10	13	.867
E2	14	21	
E3	48	36	
Mild	14	20	.259
Moderate	29	30	
Severe	29	20	
CD			
A			
A1	7	3	.437
A2	32	3	
A3	8	16	
L			
L1	8	4	.121
L2	14	5	
L3	25	13	
B			
B1	28	6	.167
B2	10	9	
B3	9	7	
Mild	11	6	.932
Moderate	15	7	
Severe	21	9	
Alb (g/L)	34.47 ± 6.97	38.2 ± 7.01	<.001*
Fasting blood glucose	5.7 ± 1.8	6.3 ± 2.2	.025*
TC (mmol/L)	4.4 ± 0.9	3.5 ± 1.0	<.001*
TG (mmol/L)	1.2 ± 0.7	1.5 ± 0.6	.013*
HDL (mmol/L)	1.3 ± 0.4	1.4 ± 0.4	.051
LDL (mmol/L)	2.4 ± 0.7	2.1 ± 0.6	.021*
ALT (U/L)	20.3 ± 40.1	16 ± 9.1	.255
AST (U/L)	23.3 ± 58.8	16.5 ± 9.1	.216
ALP (U/L)	64.3 ± 21.6	60.4 ± 11.8	.123
TBIL (umol/L)	10.5 ± 5.3	10.1 ± 6.0	.592
BUN (mmol/L)	4.6 ± 1.8	4.6 ± 2.4	.962
Cr (umol/L)	57.2 ± 13.4	58.9 ± 28.6	.589
Hb (g/L)	121.5 ± 22.7	110 ± 21.9	<.001*
PLT (×10^9^/L)	289.2 ± 95.9	299.1 ± 95.2	.46
CRP (mg/L)	24.9 ± 40.14	4.32 ± 5.35	<.001*
ESR (mm/60 min)	24.59 ± 21.48	9.56 ± 6.82	<.001*

^§^Crohn’s disease Montreal types of UC: E1 (the rectum), E2 (the left hemicolon), E3 (the extensive colon); Montreal types of CD: A1 (age of onset ≤ 16), A2 (age of onset between 17 and 40), A3 (age of onset ≥ 40); L1 (terminal ileum), L2 (colon), L3 (ileocolon), L4 (upper gastrointestinal tract); B1 (no narrow and penetrating), B2 (narrow), B3 (penetrating).

BMI, body mass index; IBD, inflammatory bowel disease; alb, albumin; TC, total cholesterol; TG, total triglycerides; HDL, high-density lipoprotein; LDL, low-density lipoprotein; ALT, alanine aminotransferase; AST, aspartate aminotransferase; ALP, alkaline phosphatase; TBIL, total bilirubin; BUN, urea nitrogen; Cr, creatinine; Hb, hemoglobin; PLT, platelet; CRP, C-reactive protein; ESR, erythrocyte sedimentation rate; UC, ulcerative colitis.

*P < .05 is considered statistically significant.

**Table 2.** Risk Factors of IBD Patients with Low Muscle Mass

Factors	Univariate Logistic Regression			Multivariate Logistic Regression		
	OR	*B*	*P*	OR	*B*	*P*
BMI	0.675-0.823	-0.294	<.001	0.659-0.935	-0.242	.007^*^
Smoking history	1.488-4.561	0.958	.001	1.433-14.219	1.507	.010^*^
Gastrointestinal surgery history	1.749-5.435	1.126	<.001	1.266-10.960	1.315	.017^*^
Parenteral manifestations	1.445-4.455	0.931	.001	0.646-5.660	0.648	.242
IBD-related complications	0.304-0.931	0.631	.027	0.198-1.697	-0.545	.32
Diagnosis (UC or CD)	1.136-3.799	0.731	.018	0.492-37.845	1.462	.187
Alb	0.886-0.963	-0.079	<.001	0.908-1.079	-0.01	.818
Fasting blood glucose	1.014-1.345	0.156	<.001	0.674-1.388	-0.033	.858
TC	0.264-0.589	-0.93	<.001	0.322-1.159	-0.493	.131
TG	1.129-3.532	0.692	.017	0.876-3.868	0.61	.107
HDL	0.986-5.368	0.833	.054			
Hb	0.965-0.990	-0.023	<.001	0.949-0.993	-0.029	.011^*^
CRP	1.038-1.122	0.076	<.001	0.994-1.132	0.059	.075
ESR	1.061-1.138	0.094	<.001	1.010-1.122	0.062	.020^*^

BMI, body mass index; IBD, inflammatory bowel disease; alb, albumin; TC, total cholesterol; TG, total triglycerides; HDL, high-density lipoprotein; Hb, hemoglobin; CRP, C-reactive protein; ESR, erythrocyte sedimentation rate, OR, odds ratio; CD, Crohn’s disease; UC, Ulcerative colitis.

*These bold values were *P *< .05, which represents statistically significant.

**Table 3.** Univariate and Multivariate Logistic Regression of Using of Steroids or Biologics or Immunomodulators Within 1 year on Low Muscle Mass

Factors	N	Univariate Logistic Regression			Multivariate Logistic Regression		
		OR	*B*	*P*	OR	*B*	*P*
Use of steroids within 1 year	62	5.167-31.415	2.545	<.001^*^	4.253-26.505	2.362	<.001^*^
Methylprednisolone	45						
Prednisone	17						
Time of using steroids within 1 year		1.186-3.157	0.66	.008^*^			
≤7 months	41						
>7 months	21						
Use of biologics within 1 year	35	2.175-15.813	1.769	<.001^*^	1.236-10.504	1.282	**.019** ^*^
Remicade	30						
Adalimumab	5						
Time of using biologics within 1 year		1.136-8.501	1.134	.027^*^			
≤7 months	20						
>7 months	15						
Use of immunomodulators within 1 year	10	0.672-15.655	1.177	.143			
Thalidomide	6						
Azathioprine	4						
Time of using immunomodulators within 1 year		0.613-7.283	0.748	.236			
≤6 months	6						
>6 months	4						

OR, odds ratio.

**Table 4.** Univariate and Multivariate Logistic Regression of Using of Hormones or Biologics or Immunomodulators Within 1 Year on Readmission Within 1 Year

Factors	N	Univariate Logistic Regression			Multivariate Logistic Regression		
		OR	*B*	*P*	OR	*B*	*P*
Use of steroids within 1 year	62	2.363-8.663	1.509	<.001^*^	2.096-8.017	1.411	**<.001** ^*^
Methylprednisolone	45						
Prednisone	17						
Time of using steroids within 1 year		1.135-2.190	0.455	.007^*^			
≤7 months	41						
>7 months	21						
Use of biologics within 1 year	35	1.154-5.263	0.902	.02	0.690-3.585	0.453	<.001 ^*^
Remicade	30						
Adalimumab	5						
Time of using biologics within 1 year		1.211-3.612	0.738	.008^*^			
≤7 months	20						
>7 months	15						
Use of immunomodulators within 1 year	10	0.116-1.827	-0.778	.27			
Thalidomide	6						
Azathioprine	4						
Time of using immunomodulators within 1 year		0.732-23.955	-0.847	.107			
≤6 months	6						
>6 months	4						

OR, odds ratio.

*P < .05 is considered statistically significant.

**Table 5.** Relationship of UC, CD Patients and Readmission Within 1 Year in Mild, moderate, and Severe Subgroups

	Readmission Within 1 Year	Without Readmission Within 1 Year	*P*
UC			
Mild group			
With low muscle mass	7	7	.027 ^*^
Without low muscle mass	3	17	
Moderate group			
With low muscle mass	20	9	.047 ^*^
Without low muscle mass	13	17	
Severe group			
With low muscle mass	18	11	<.001 ^*^
Without low muscle mass	3	17	
CD			
Mild group			
With low muscle mass	6	5	<.001 ^*^
Without low muscle mass	0	6	
Moderate group			
With low muscle mass	11	4	<.001 ^*^
Without low muscle mass	1	6	
Severe group			
With low muscle mass	15	6	<.001 ^*^
Without low muscle mass	3	6	

UC, ulcerative colitis; CD, Crohn’s disease.

^*^ *P *< .05 is considered statistically significant.
